# Fabrication of Multiple Parallel Microchannels in a Single Microgroove via the Heating Assisted MIMIC Technique

**DOI:** 10.3390/mi13030364

**Published:** 2022-02-25

**Authors:** Dengying Zhang, Wenqiang Xing, Weiren Li, Shengming Liu, Yanli Dong, Lichun Zhang, Fengzhou Zhao, Jun Wang, Zheng Xu

**Affiliations:** 1 Institute of Optoelectronics Technology, Beijing Jiaotong University, Beijing 100044, China; 2 Beijing Solar Power Research Institute Co., Ltd., Beijing 101102, China; wangjun_jiechen@126.com; 3 College of Physics and Optoelectronic Engineering, Ludong University, Yantai 264025, China; xqzl1996@gmail.com (W.X.); oneselfall@outlook.com (W.L.); shengmingliu123@163.com (S.L.); ChengyueDongyl@163.com (Y.D.); phyzlc@163.com (L.Z.); fzzhao@ldu.edu.cn (F.Z.); 4 Key Laboratory of Luminescence and Optical Information (Beijing Jiaotong University), Ministry of Education, Beijing 100044, China

**Keywords:** double-microchannel phenomenon, parallel microchannels, capillary action, PDMS guiding mold

## Abstract

For the first time, multiple parallel microchannels in a single microgroove have been fabricated by the heating-assisted micromolding in capillaries technique (HAMIMIC). Microchannel development, cross-sectional shape, and length were all explored in depth. The factors affecting the cross-sectional shape and length of the double-microchannel were also discussed. Finally, a special-shaped PDMS guiding mold was designed to control the cross-sectional shape and length of multiple parallel microchannels for controlled growth. The HAMIMIC technique provides a low-cost, straightforward, and repeatable way to create multiple parallel microchannels in a single microgroove, and will promote the progress of bifurcated vessels and thrombus vessels preparation technology.

## 1. Introduction

In recent decades, microchannels have attracted tremendous attention due to their wide applications in various fields such as biomedicine [1,2,3,4,5,6,7,8,9,10,11,12,13], optics [14,15], and microfluidic [16,17,18]. Correspondingly, there are already many approaches to fabricate microchannels, such as a glass cover on silicon [19], sacrificial layer [20], wafer bonding [21], sealing [22], porous silicon [23], buried channel technology [24], electric field-assisted capillarity [25], 3D printing [26], and soft lithography [27,28,29], etc. Compared with other approaches, soft lithography is widely used for applications ranging from simple microchannel fabrication to the creation of micropatterns onto a surface. It can also be used within a microfluidic channel, due to its remarkable qualities, such as ease of operation, low cost, high fidelity, and high resolution. A soft polymeric mold, such as a polydimethylsiloxane (PDMS) duplicate of an original hard master, is used in soft lithography. One type of soft lithography is micromolding in capillaries (MIMIC) [29].

Traditional microchannels have a rectangular cross-section with high surface-to-volume ratio [30,31], which cannot meet the needs of some special applications. For example, if we want to examine the relevant characteristics of blood flow in blood vessels, it is necessary to use circular microchannels that mimic human blood vessels, as the rectangular microchannels will result in an inaccurate result. Until now, several strategies have been proposed to fabricate microchannels with a circular cross-section. The typical approach is to bond two semi-circular PDMS microchannels together [32]. However, the misalignment during the bonding process impeded the application of this approach. The second approach involves covering the rectangular tubes with liquid PDMS on their surface [33]. However, producing microchannels with a diameter of tens of microns or less with this technology is difficult. Another method is to distort a partially cured PDMS with expanded air pressure [34]. This approach can produce circular microchannels when a semi-circular PDMS mold is utilized. However, the experimental setup employed in this method is complex, particularly the use of an Arduino board, which makes those lacking experience back down. As a result, a simple and effective approach for producing circular microchannels within tens of microns still has to be developed.

Recently, our group developed a new approach called the heating assisted MIMIC technique (HAMIMIC) to fabricate circular cross-section microchannels. This technique combines the MIMIC method with subsequent heat treatment [35], and demonstrates the ability to create microchannels with various cross-sectional shapes and diameters less than 20 μm. The cross-section and size of microchannels can be controlled by changing the shape and size of the microgrooves in the PDMS mold. When the widths of the microgrooves in the PDMS mold increased to 40 μm, two parallel microchannels formed in a single microgroove. However, the isolation wall between two parallel microchannels was not precisely in the middle, producing two parallel microchannels with different diameter. In order to obtain parallel microchannels of the same size, we designed a new PDMS mold with a special cross-section and obtained two identical parallel microchannels in a single microgroove. Following this, three parallel microchannels with the same cross-section were also fabricated in a single microgroove.

## 2. Materials and Methods

### 2.1. Materials and Reagents

The S1813 Microposit photoresist and Shipley Type P Microposit thinner were purchased from Shipley (Marlborough, MA, USA). Polydimethylsiloxane (PDMS, Sylgard 184) and the curing agent were purchased from Dow Corning Corporation (Midland, MI, USA). The AZ9260 photoresist and AZ400K developer were purchased from AZ Electronic Materials (Somerville, NJ, USA). The K_9_ glass substrates were purchased from Nantong Yinxing Optical Co., Ltd. (Nantong, China). The lithography mask was purchased from the Fiftieth Research Institute of China Electronic Technology Group Corporation (Nanjing, China). All other chemicals were of analytical grade and were used without further purification.

### 2.2. Preparation of Masters and PDMS Molds

In order to obtain ordinary PDMS molds and PDMS guiding molds, firstly, the ordinary masters and the guiding masters were prepared.

Preparation of masters. The ordinary masters were prepared by photolithography in the AZ9260 photoresist. The AZ9260 photoresist layers with different thicknesses were spin-coated on a 3-inch silicon wafer with different rotating speeds and spin coating time using a Laurell WS-650Mz-23NPPB spin coater. The photoresist soft-bake was performed for 10 min on a hotplate (95 °C). The photoresist development was carried out with AZ400K developer (DI:AZ400K = 4:1) after UV exposure (Midas MDA-400M, about 1050 mJ/cm^2^, adjusting the exposure dose according to the thickness of photoresist). The dome-shaped master was created using a thermal reflow procedure at 140 °C for 30 min. The guiding masters were prepared by photolithography in the S1813 photoresist. The S1813 photoresist was spin-coated for 40 s on a 3-inch silicon wafer at 1200 rpm, and then heated to 90 °C for 5 min to cure the film. The above operation was repeated three times to obtain a film with a thickness of ~3.4 μm. The photoresist development was carried out in a sodium hydroxide solution with a volume concentration of 5‰ after UV exposure (Midas MDA-400M, 110 mJ/cm^2^).

Preparation of PDMS molds. The PDMS molds were fabricated by casting the mixture of base and curing agent (weight ratio 10:1) on the target masters. The PDMS polymer was then left to cure for 24 h. After the PDMS molds had fully cured, they were peeled away from the masters.

### 2.3. HAMIMIC Method

**Figure 1:**
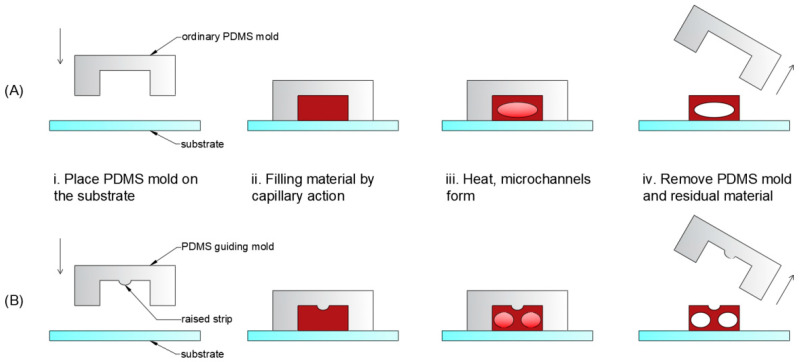
Schematic of fabrication process of the single microchannel (**A**) and the parallel double-microchannels (**B**) in a single microgroove, which consists of four main steps: (**i**) Place PDMS mold on the substrate; (**ii**) Filling material by capillary action; (**iii**) Heat, microchannels form; (**iv**) Remove PDMS mold and the residual material.

Figure 1 depicts a schematic of the fabrication procedure. The K_9_ glass substrate was cleaned using ultrasonic cleaner in ethylalcohol and deionized water for 5 min each, then dried with nitrogen flow. The PDMS mold was then used to create a microchannel cavity on the K_9_ glass substrate. To make polymer microchannels, Shipley Microposit S1813 photoresist was diluted with Shipley Type P Microposit thinner at a 1:2 volume ratio (photoresist: thinner). When the photoresist liquid dropped to one end of the microchannels, it filled the microchannels spontaneously under the capillary action. After an hour, the samples were cured on a hotplate for 40 min at 120 °C. The samples were then cooled to room-temperature. After the PDMS mold and residual material were removed, the polymer microchannels were created on the substrate. It is worth noting that the PDMS guiding mold has a raised strip on top of the microgroove, but the ordinary PDMS mold does not.

### 2.4. Characterization Measurements

The geometric characteristics of the polymer microchannels were investigated using the Leica DM8000M optical microscope and the Hitachi SU8010 field emission scanning electron microscope (FE-SEM), respectively.

## 3. Results and Discussion

### 3.1. Single Microchannel

**Figure 2:**
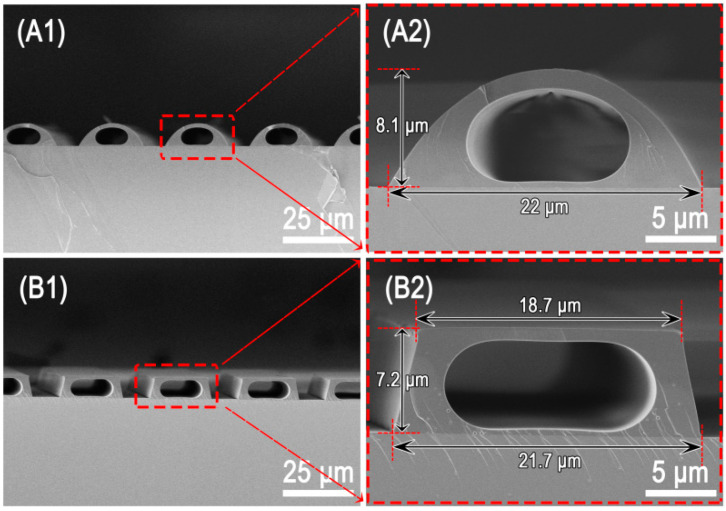
Microchannels fabricated with different PDMS molds: (**A1**,**A2**) microchannels with capsule-shaped cross-section inside the spherical coronal microstructures; (**B1**,**B2**) microchannels with capsule-shaped cross-section inside the trapezoid-microstructures.

Figure 2 presented two typical instances of microchannels fabricated with various PDMS molds using the procedure depicted in Figure 1A. The cross-section of the microchannels can be modified by adjusting the size and cross-sectional profile of microgrooves in the PDMS molds, as shown in Figure 2A1,A2,B1,B2. The microchannel structure with capsule cross-section can be obtained regardless of whether the microgroove’s cross-section form is semicircular or trapezoidal. By changing the width and height of the microgrooves in the PDMS mold, we were able to efficiently build a microchannel structure with a circular cross-section [35].

### 3.2. Double Microchannels

**Figure 3:**
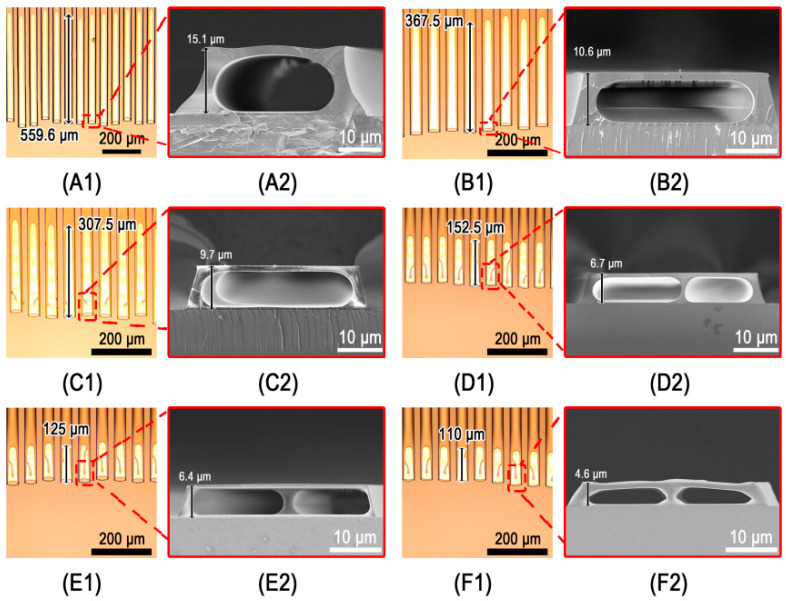
Microchannels fabricated in trapezoid microgrooves with a bottom width of about 40 μm and different heights: (**A1**,**A2**) 15.1 μm; (**B1**,**B2**) 10.6 μm; (**C1**,**C2**) 9.7 μm; (**D1**,**D2**) 6.7 μm; (**E1**,**E2**) 6.4 μm; (**F1**,**F2**) 4.6 μm; (**A1**–**F1**) the microscope images of microchannels; (**A2**–**F2**) the cross-sectional images of microchannels corresponding to (**A1**–**F1**).

**Figure 4:**
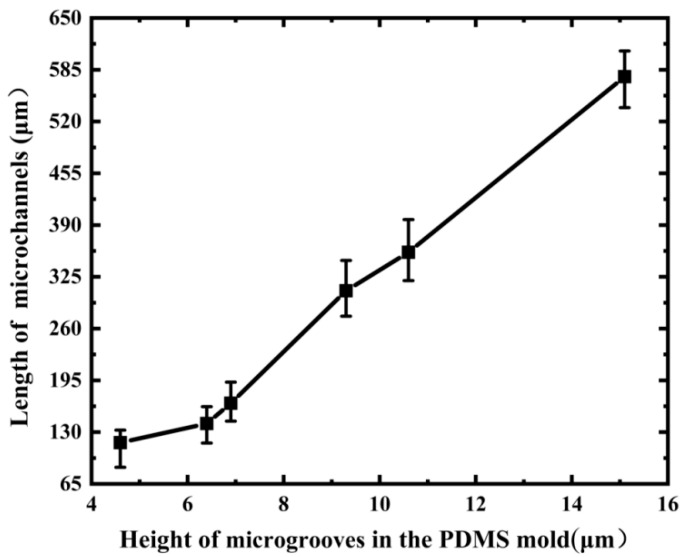
Length of microchannels versus the height of 40 μm wide microgrooves in the PDMS mold.

In prior research, we studied the influence of a PDMS microgroove with a width of less than 20 μm on the development of microchannels, but we did not explore the effect of a wider microgroove on microchannel preparation. Therefore, when the microgroove width reached around 40 μm, we investigated the formation of microchannels as the microgroove height decreased, as shown in Figure 3. Only one microchannel formed in a microgroove when the height of the microgroove was more than 10.6 μm, as shown in Figure 3A1,A2,B1,B2. When the height of microgrooves was reduced to 9.7 μm, a shorter bifurcate structure appeared in the microchannels, as illustrated in Figure 3C1,C2. The longer bifurcate structure created when the microgroove height was lowered to 6.7 μm, is seen in Figure 3D1,E1,F1. Two parallel microchannels in the same microgrooves were also formed, as shown in the cross-sectional images of Figure 3D2, E2, F2. The isolation wall between the two parallel microchannels, on the other hand, was usually not at the center of the microgrooves because the microchannels were frequently of different size. Moreover, the isolation wall’s end was not situated at the end of the microchannels (see Figure 3C1,D1,E1), preventing the long microchannels from being divided into two uniform and parallel microchannels. Because the microchannel wall becomes very thin and easy to rupture during the PDMS mold removing process when the microgroove width reaches around 80 μm, obtaining microchannels in either single microchannel or double microchannels becomes challenging. We believe that it is achievable if the appropriate material is used. Figure 4 demonstrates the relationship between the length of microchannels and the height of 40 μm wide microgrooves in the PDMS mold. Figure 3 and Figure 4 show that the length of microchannels reduced as the microgroove height lowered, which was consistent with the findings of the previous study [35]. The formation mechanism of the microchannels can be easily understood on a qualitative level. As is well known, the smaller the capillary radius, the stronger the capillary force generated by the liquid. Therefore, when the microgroove width is held constant, the capillary force increases as the microgroove height decreases. When photoresist backflow occurs in S1813, the capillary force in the microgroove prevents photoresist backflow. The stronger the capillary force, the more photoresist backflow resistance and the shorter the microchannels that eventually emerge.

### 3.3. Mulitiple Parallel Microchannels

**Figure 5:**
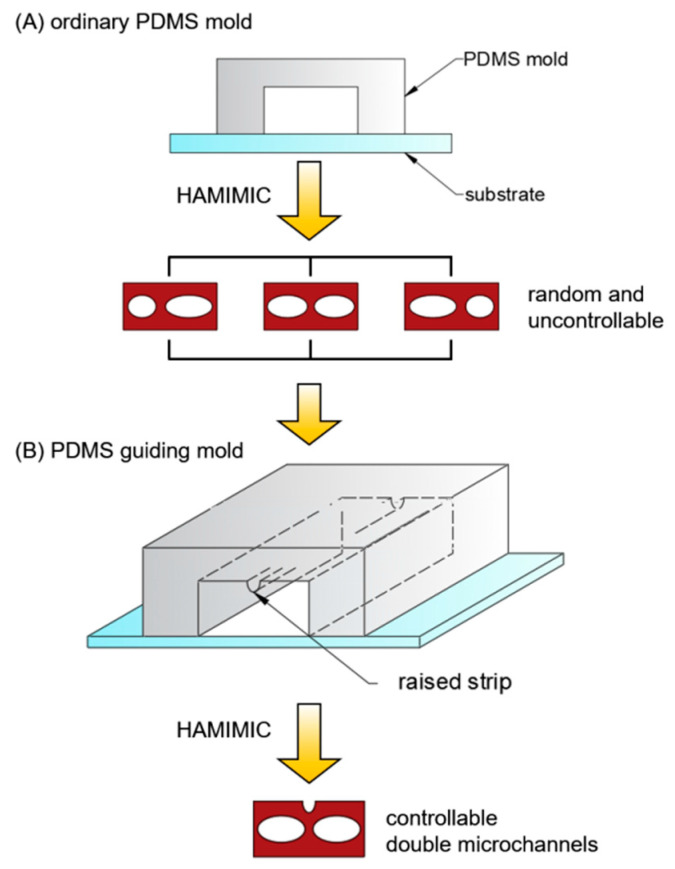
Schematic illustration of fabricating two parallel microchannels with ordinary PDMS mold (**A**) and PDMS guiding mold (**B**).

The schematic illustration of constructing two parallel microchannels with the standard PDMS mold and the PDMS guiding mold, respectively, is shown in Figure 5. The two parallel microchannels in the same microgroove can be obtained using an ordinary PDMS mold by simply changing the height of the microgroove, as shown in Figure 3D1,D2,E1,E2,F1,F2. However, we discovered that the sizes of the left and right microchannels made using ordinary PDMS molds were often inconsistent, arbitrary, and unpredictable, as shown in Figure 3D1,D2,E1,E2,F1,F2, and Figure 5A. Therefore, we designed the PDMS guiding mold to obtain two parallel microchannels of nearly equal size, in a single microgroove which has a raised strip in the middle of the top, as shown in Figure 5B. Two parallel microchannels were obtained in a single microgroove using the fabrication procedure indicated in Figure 1B.

**Figure 6:**
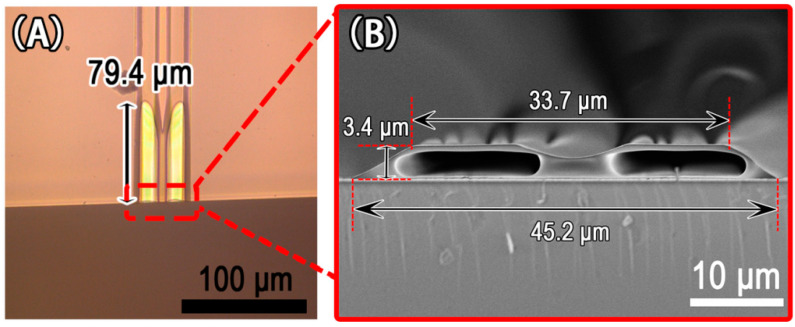
Images of two parallel microchannels fabricated with a PDMS guiding mold: (**A**) surface microscope photograph; (**B**) SEM image of the cross-section.

Images of two parallel microchannels fabricated with a PDMS guiding mold are shown in Figure 6. The two parallel microchannels had a length of at least 79.4 μm (see Figure 6A). Figure 6B depicts the cross-sectional view of two parallel microchannels made using a trapezoidal microgroove with upper and bottom widths of 33.7 μm and 45.2 μm, respectively, and a height of 3.4 μm. Pre-cutting the glass substrate on the back side, without breaking it in advance, can be used when preparing such a sample. After the microchannels were produced, the glass substrate was broken at the pre-cut line to obtain the cross-section of two parallel microchannels, as shown in Figure 6B. The arc in the middle of the cross-section of the two parallel microchannels corresponded with the raised strip at the top of the microgroove in the PDMS guiding mold (see Figure 5B). These findings demonstrated that the HAMIMIC technology can controllably construct parallel microchannels in a single microgroove using guiding mold.

**Figure 7:**
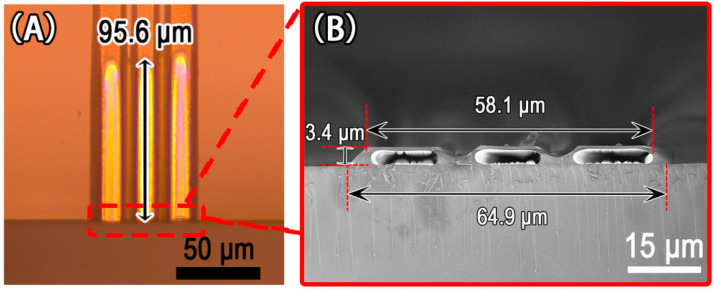
Images of three parallel microchannels fabricated with a PDMS guiding mold: (**A**) surface microscope photograph; (**B**) SEM image of the cross-section.

Figure 7 exhibits the images of three parallel microchannels fabricated with a PDMS guiding mold. The length of the three parallel microchannels was approximately 96.5 μm, as shown in Figure 7A. The cross-section of the three parallel microchannels had top and bottom widths of 58.1 μm and 64.9 μm, respectively, and a height of 3.4 μm, as shown in Figure 7B. Our approach can readily prepare several parallel microchannels in a single microgroove, as demonstrated by this experimental result. Thermodynamics and kinetics theory of MIMIC [29,36] can be used to understand the capillary action mechanism in our experiments. The liquid filled the capillaries in the MIMIC process, lowering the free energy at the solid–vapor and solid–liquid interfaces. When the heating process began, the polymer–colloidal interface in the PDMS microchannels began to recede in order to meet the requirements of the increased surface free energy. However, only the polymer in the microchannel’s center receded with time, whereas the polymer close to the inner wall of the microgroove adhered to the inner wall of the microgroove without receding, and the thin-walled microchannels formed. The isolation wall between the multiple parallel microchannels was the result of gravity effect and surface tension. Elsewhere, the mechanism of microchannel development has been elucidated [35]. It is worth noting that photolithography is not required for our parallel microchannels fabrication procedure if a suitable master or PDMS guiding mold is obtained. As a result, it is quite simple to make several parallel microchannels based on actual demands.

## 4. Conclusions

In summary, we developed the novel technology HAMIMIC to fabricate multiple parallel microchannels in a single microgroove. By adjusting the size and cross-section profile of the microgrooves in the PDMS mold, the cross-section shape of the microchannels may be easily controlled. In the experiment, we used a PDMS mold with a microgroove width more than 40 μm and discovered that when the microgroove height was reduced to 6.7 μm, a double-microchannel formed in a single microgroove. However, because the two microchannels generated in this situation were irregular and unmanageable, the PDMS guiding mold was developed to create controllable parallel microchannels. Using the PDMS guiding mold, samples of double and triple parallel microchannels were obtained in a single microgroove. It is the first time that several parallel microchannels have been produced in a single microgroove on a PDMS mold. The HAMIMIC technology provides a low-cost, straightforward, and repeatable way to create multiple parallel microchannels in a single microgroove, and will promote the progress of bifurcated vessel and thrombus vessel preparation technology.

## Data Availability

All experimental data are available upon reasonable request from the corresponding authors via email.
